# Increased Retinol Levels in Patients with Cardiac Surgery-Associated Acute Kidney Injury—A Prospective Single-Center Exploratory Study

**DOI:** 10.3390/nu18121921

**Published:** 2026-06-13

**Authors:** Anna Olasińska-Wiśniewska, Ewelina Swora-Cwynar, Tomasz Urbanowicz, Marta Karaźniewicz-Łada, Julia Kerner, Anna Siemiątkowska, Agnieszka Dobrowolska, Bartłomiej Perek, Marek Jemielity

**Affiliations:** 1 Department of Cardiac Surgery and Transplantology, Poznan University of Medical Sciences, 61-848 Poznan, Poland; turbanowicz@ump.edu.pl (T.U.); bperek@ump.edu.pl (B.P.); marek.jemielity@usk.poznan.pl (M.J.); 2 Department of Gastroenterology, Dietetics and Internal Medicine, Poznan University of Medical Sciences, 60-355 Poznan, Poland; eswora@ump.edu.pl (E.S.-C.); agdob@ump.edu.pl (A.D.); 3 Department of Physical Pharmacy and Pharmacokinetics, Poznan University of Medical Sciences, 60-806 Poznan, Poland; mkaraz@ump.edu.pl (M.K.-Ł.); julia.kerner@ump.edu.pl (J.K.); asiemiatkowska@ump.edu.pl (A.S.)

**Keywords:** cardiac surgery, acute kidney injury, retinol, vitamin metabolism

## Abstract

Background: Acute kidney injury (AKI) is a frequent and prognostically important complication of cardiac surgery, yet early risk stratification remains challenging. The purpose of this prospective exploratory study was to determine whether preoperative vitamin levels differ in patients who develop cardiac surgery-associated AKI. Methods: Consecutive patients scheduled for cardiac surgery due to coronary artery disease and/or severe aortic stenosis between October 2024 and July 2025 were included. Fourteen patients (16.1%) had preoperative eGFR below 60 mL/min. Preoperative serum levels of vitamin A (retinol), vitamin E (α-tocopherol), and vitamin D (25-hydroxyvitamin D3) were measured. Results: A total of 87 patients (72 males (82.8%) with a median (Q1–Q3) age of 66 (61.5–71) years) were included in the study. Cardiac surgery-associated AKI occurred in 36 (41.4%), as a mild and transient impairment, with only two patients with a more severe stage requiring temporary renal replacement therapy. Patients who developed AKI had significantly higher preoperative retinol levels (*p* = 0.046). Retinol concentrations correlated positively with preoperative creatinine (Spearman’s rho 0.321, *p* = 0.002), postoperative day 0 creatinine (Spearman’s rho 0.333, *p* = 0.002), and postoperative day 1 creatinine (Spearman’s rho 0.268, *p* = 0.012), and negatively with preoperative eGFR (Spearman’s rho −0.288, *p* = 0.007). Tocopherol and 25(OH)D3 did not differ significantly between subgroups. No difference in vitamin levels was observed between patients with and without diabetes. Conclusions: Increased preoperative retinol levels were associated with cardiac surgery-associated AKI and correlated with perioperative renal dysfunction. Retinol may reflect impaired kidney handling of retinol and identify increased renal vulnerability in cardiac surgery patients. Retinol may represent a hypothesis-generating biomarker of cardiac surgery-associated AKI risk that warrants confirmation in larger cohorts.

## 1. Introduction

Acute kidney injury (AKI) is a frequent complication of cardiac surgery and affects approximately 5% to 45% of patients [1]. It is associated with increased mortality, the need for temporary or permanent renal replacement therapy (RRT), prolonged intensive care unit stay, and longer hospitalization [2]. Even mild AKI may adversely affect short-term recovery. RRT is necessary in 1–5% of patients with postoperative AKI and is associated with worse prognosis in the short- and long-term follow-up [3]. Notably, even reversible postoperative kidney injury is also linked to an increased risk of chronic kidney disease (CKD) and long-term morbidity and mortality.

The pathophysiology of AKI is complex and multifactorial. Depending on baseline patient characteristics, surgical procedure, and perioperative complications, the severity of kidney injury may vary substantially. Several mechanisms are involved in the development of AKI, including inflammation, oxidative stress, hypoperfusion, ischemia–reperfusion injury, neurohormonal responses, labile iron-mediated toxicity, and exposure to nephrotoxic agents [3,4,5,6].

Numerous perioperative factors [7] have been identified as predictive for AKI (Supplementary Table S1). They may be classified as patient-related, such as age, female sex, pre-operative nutrition status; co-morbidities, including diabetes mellitus (DM), chronic obstructive pulmonary disease (COPD), peripheral arterial disease (PAD), heart failure (HF); procedural factors such as cardiopulmonary bypass (CPB) use, hemodilution, type of surgery; and postoperative such as low cardiac output and sepsis [3,8,9]. Cardiac surgery-associated AKI is linked to a higher incidence of other postoperative complications, including infection, prolonged intensive care unit stay, stroke, and heart failure [10]. Patients with preoperative impaired kidney function are at a particularly higher risk of adverse postoperative outcomes.

Chronic kidney disease (CKD) is characterized by exaggerated inflammatory response that contributes to increased morbidity and mortality. Inflammation is also implicated in the pathogenesis of coronary artery disease (CAD) and aortic stenosis (AS). Both types of heart diseases often co-exist with varying degrees of kidney dysfunction, reflecting their shared epidemiological and pathophysiological mechanisms, impaired peripheral blood flow, and a proinflammatory milieu [11]. Alterations in vitamin metabolism have recently emerged as an important issue in patients with CKD [12]. Vitamins E and A have anti-inflammatory and antiapoptotic effects, and vitamin D has been postulated to modulate inflammatory pathways [13,14,15,16]. Dysregulation of vitamin status has been associated with worse postoperative outcomes and higher complication rates [17].

The aim of the study was to determine whether preoperative levels of liposoluble vitamins differ in patients presenting with cardiac surgery-associated AKI and may be useful in the preoperative prediction of AKI.

## 2. Materials and Methods

This prospective exploratory study included consecutive patients admitted to the cardiac surgery department between October 2024 and July 2025. The exclusion criteria were non-open-chest procedures, storage diseases, and isolated surgeries unrelated to CAD and/or AS. None of the patients followed a restrictive diet or used vitamin supplements systematically. Long-term vitamin supplementation was an exclusion criterion. Demographic and clinical data were collected at admission.

The inclusion of an age-matched healthy control group was not feasible, as such individuals would not undergo surgery, and therefore, the occurrence of cardiac surgery-associated AKI could not be assessed in this group.

Arterial hypertension (HA) was defined as systolic blood pressure of at least 140 mmHg and/or diastolic blood pressure of at least 90 mmHg, or use of antihypertensive medication. Diabetes mellitus (DM) and impaired glucose tolerance (IGT) were documented based on the medical history and medication use. Chronic obstructive pulmonary disease (COPD) was recorded according to the use of inhaled therapy. Smoking history was also obtained.

Preoperative echocardiography was performed using a standardized protocol. Valvular functions were assessed, including aortic stenosis severity based on peak and mean transvalvular gradients and aortic valve area, valvular insufficiencies, left ventricular contractility, left ventricular ejection fraction, and aortic root enlargement.

Coronary angiography was performed in all patients to assess the coronary atherosclerotic burden. Any history of percutaneous coronary angioplasty (PCI) was recorded.

Blood samples were collected for blood cell counts, serum creatinine, and N-terminal prohormone of brain natriuretic peptide (NT-proBNP).

Estimated glomerular filtration rate (eGFR) was calculated using the Modification of Diet in Renal Disease (MDRD) Study equation. Cardiac surgery-associated AKI [18] was defined according to Kidney Disease: Improving Global Outcomes (KDIGO) criteria, as an increase in serum creatinine (SCr) by at least 0.3 mg/dL within 48 h, an increase to at least 1.5 times baseline within 7 days, or urine output below 0.5 mL/kg/h for 6 h. Temporary kidney replacement therapy was recorded.

Blood samples for vitamin A, E, and D analysis were collected at patients’ admission to the hospital, after a 6 h fasting period, and centrifuged at 3000× *g* for 15 min at 4 °C. Serum was aliquoted and stored at −80 °C until analysis. We focused on fat-soluble vitamins because they are involved in several biological pathways relevant to the development of cardiac surgery-associated acute kidney injury, including oxidative stress, systemic inflammation, and endothelial dysfunction.

### 2.1. Analysis of Vitamins by UPLC-MS/MS Method

The analysis was carried out using a Shimadzu UPLC Nexera system coupled to an LCMS-8030 detector (Shimadzu Co., Kyoto, Japan). The separation of three analytes: vitamin A (retinol), vitamin E (α-tocopherol), and vitamin D (25-hydroxyvitamin D3, 25(OH)D3), along with their internal standards (ISs: retinol-C13, α-tocopherol-d6, 25-hydroxyvitamin D3-d6), was achieved on a Kinetex C18 column (100 × 2.1 mm; 2.6 μm) (Torrance, CA, USA). The column temperature was maintained at 40 °C. The mobile phase consisted of a methanol–water mixture, both containing 0.1% (*v*/*v*) formic acid, delivered using gradient elution at a flow rate of 0.45 mL/min. The injection volume was set to 10 μL. Eluent was introduced into the MS interface using positive electrospray ionization (ESI+) mode. The electrospray capillary voltage was set at 4.5 kV. The MS interface parameters included a desolvation line temperature of 230 °C, a heat block temperature of 400 °C, and an interface temperature of 350 °C. Nitrogen was used as the drying gas and as the nebulizing gas with flow rates of 15 and 2 L/min, respectively. The multiple reaction monitoring (MRM) mode was used to monitor the specific transitions of the analytes. The most sensitive mass transition was observed from *m*/*z* 269.2 to 93.0 for retinol, from *m*/*z* 431.3 to 165.1 for α-tocopherol, and from *m*/*z* 401.2 to 383.3 for 25-hydroxyvitamin D3. For their ISs, the following mass transitions were monitored: 272.2 ⟶ 84.0 for retinol-C13, 437.4 ⟶ 171.2 for α-tocopherol-d6, and 407.2 ⟶ 389.4 for 25-hydroxyvitamin D3-d6. Calibration curves of the analytes were prepared in concentration ranges of 0.02–2 mcg/mL for retinol, 0.5–20 mcg/mL for α-tocopherol and 5–125 ng/mL for 25-hydroxyvitamin D3. The intra- and inter-day accuracy of the method, expressed as the relative error, was <15%. The intra- and inter-assay precision, expressed as the relative standard deviation, was <10%.

Patient samples were prepared by adding 20 μL of the ISs solution and 4 μL of 0.1% 2,6-di-tert-butyl-4-methylphenol (BHT) in methanol to 36 μL of patient serum. The mixture was vortexed for 10 s, followed by the addition of 90 μL of 0.1% BHT in methanol to induce protein precipitation, and vortexed again for 10 s. Subsequently, the samples were mixed for 5 min at 750 rpm and 4 °C, then centrifuged for 5 min at 18,000× *g* (4 °C). The resulting supernatant was transferred to a clean glass vial and directly injected into the UPLC–MS/MS system. Patient samples for the analysis were prepared in duplicate, and two independent measurements were performed for each sample.

Reference values for vitamin concentrations are as follows: retinol, 0.3–0.7 mcg/mL; tocopherol, 5–20 mcg/mL; and vitamin D (25(OH)D3), 30–50 ng/mL.

### 2.2. Statistical Analysis

The Shapiro–Wilk test was applied to assess data distribution. Continuous variables were presented as medians (Q1–Q3) and were compared using the Mann–Whitney U tests for two groups or the Kruskal–Wallis for multiple subgroups. Categorical variables were expressed as frequencies (%) and were analyzed using the chi-squared test or Fisher’s exact test, as appropriate. Spearman’s rank correlation coefficient was used to evaluate the association between retinol levels and preoperative and postoperative kidney function parameters. Statistical analysis was performed using JASP software (JASP Team, 2025; The Netherlands, Amsterdam, Version 0.19.3) and *p* < 0.05 was considered statistically significant.

The project was approved by the Bioethics committee of the Poznan University of Medical Sciences, Poznan, Poland (No 272/2021 dated 8 April 2021 and No 151/25 dated 3 April 2025). This study was performed in line with the principles of the Declaration of Helsinki. Informed consent was obtained from all individual participants included in the study.

## 3. Results

### 3.1. Study Outcomes

**Figure 1:**
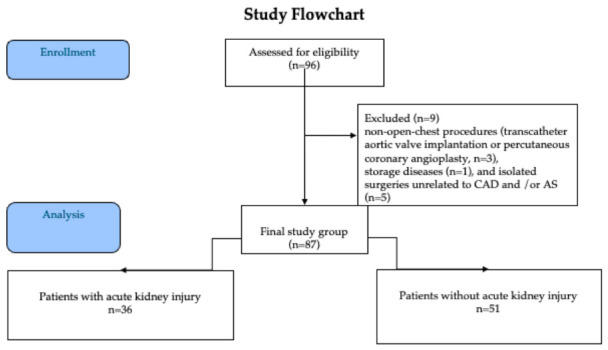
Study flowchart.

The analysis included 96 patients who were admitted for cardiac surgery (Figure 1). Nine patients met the exclusion criteria: three did not undergo open-chest cardiac surgery, one had a storage disease, and five underwent procedures other than coronary artery bypass grafting (CABG) and/or aortic valve replacement (AVR). The final study group comprised 87 patients, including 72 males (82.8%), with a median (Q1–Q3) age of 66 (61.5–71) years. Sixty-five patients were operated on due to severe CAD, 18 due to severe AS, and four underwent a combined CABG + AVR.

The majority of patients presented with cardiovascular risk factors, including arterial hypertension (*n* = 73, 83.9%), diabetes mellitus or IGT (*n* = 32, 36.8%), COPD or asthma requiring inhaled medication (*n* = 7, 8%), and a history or cardiovascular disease, including previous myocardial infarction (*n* = 18, 20.7%), previous PCI (*n* = 12, 13.8%), stroke or transient ischemic attack (TIA) (*n* = 6, 6.9%), and peripheral artery disease (PAD) (*n* = 14, 16.1%). In addition, 14 patients (16.1%) had preoperative eGFR below 60 mL/min. Baseline demographic and clinical characteristics are summarized in Table 1.

Postoperative AKI, defined as an increase in serum creatinine of more than 0.3 mg/dL within 48 h, or to more than 1.5 times baseline, occurred in 36 patients (41.4%). In 34 of these patients, AKI was mild and transient, whereas 2 required temporary RRT. No patient required permanent RRT.

Vitamin 25(OH)D3 deficiency was the most common in the study population, accounting for 66 patients (75.9%), while only 21 patients (24.1%) had higher levels. Retinol deficiency was observed in 2 patients; the majority (60.9%, *n* = 53) had values within the normal range, and 36.8% (*n* = 32) had values above the normal range. Tocopherol levels were within normal values in 88.5% (*n* = 77), while 10 patients (11.5%) showed levels that were excessive.

**Figure 2:**
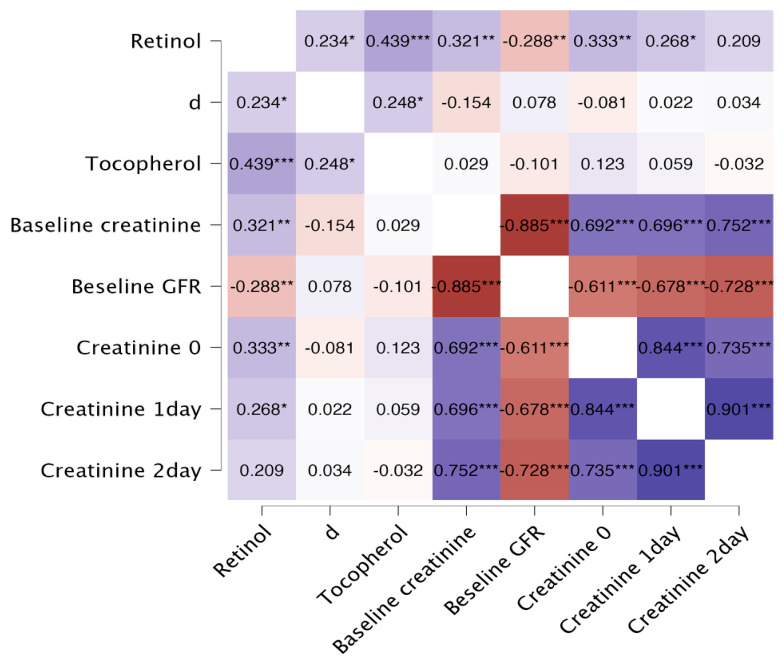
Correlations between vitamins and perioperative creatinine levels. * *p* < 0.05, ** *p* < 0.01, *** *p* < 0.001. Blue colors correspond to positive correlation coefficients, red colors correspond to negative correlation coefficients. The saturation of colors reflects the absolute value of the correlation coefficient.

Vitamin concentrations were statistically significantly higher in the AKI subgroup; however, only retinol levels differed significantly between subgroups (*p* = 0.046) (Table 1). Retinol concentrations correlated positively with preoperative creatinine (Spearman’s rho 0.321, *p* = 0.002), postoperative day 0 creatinine (Spearman’s rho 0.333, *p* = 0.002), and postoperative day 1 creatinine (Spearman’s rho 0.268, *p* = 0.012), and negatively with preoperative eGFR (Spearman’s rho −0.288, *p* = 0.007). The association with postoperative day 2 creatinine did not reach statistical significance (Spearman’s rho 0.209, *p* = 0.053) (Figure 2).

Two patients who required temporary RRT had the highest retinol levels compared to those with AKI who did not require RRT and with patients without AKI (*p* = 0.026, 1.22 (1.05–1.38) mcg/mL vs. 0.63 (0.54–0.77) mcg/mL vs. 0.56 (0.45–0.72) mcg/mL).

### 3.2. Subdivision into Diabetic and Non-Diabetic Patients

Given that several studies have highlighted the association between diabetic nephropathy and alterations in vitamin concentrations, we analyzed vitamin status in the relevant subgroups. We did not reveal differences between subgroups with and without DM, with or without AKI (Table 2).

### 3.3. Sex-Stratified Analysis

Male dominance was observed in our study population. Therefore, we performed a sex-stratified analysis to assess whether sex could account for the variance in vitamin concentrations.

In linear regression analysis, sex was significantly associated with preoperative 25(OH)D3 concentrations (unstandardized β = −12.1, (95% CI: −21.4–−2.9), *p* = 0.011), indicating lower vitamin D in women than in men. Sex alone explained approximately 7% of the variance in 25(OH)D3 concentrations (R^2^ = 0.074, adjusted R^2^ = 0.063). Sex was not significantly associated with serum retinol concentrations (unstandardized β = 0.02, 95% CI −0.11 to 0.14, *p* = 0.759) and had an almost negligible impact on the variance in its concentration (R^2^ = 0.001, adjusted R^2^ = −0.011). Tocopherol concentration was not significantly associated with sex (unstandardized β = −3.0 mcg/mL, 95% CI −6.3 to 0.3, *p* = 0.077), with sex accounting for only a small proportion of the variance in its concentration (R^2^ = 0.036, adjusted R^2^ = 0.025).

## 4. Discussion

Our study demonstrated that patients who developed cardiac surgery-associated AKI had higher preoperative retinol levels than those without the complication. Moreover, retinol correlated with both preoperative and postoperative creatinine levels, suggesting a close relationship between vitamin A status and renal function. Notably, serum retinol concentrations within the higher reference range may still correlate with AKI, suggesting that even subclinical variation in vitamin A status may be clinically relevant. Tocopherol levels were also high in the AKI group; however, the difference was not statistically significant.

Vitamin A and its metabolites are essential for human health and play important roles in immune function, cell differentiation, adipose tissue and glucose metabolism, bone mineralization, and nocturnal vision [13]. Vitamin A is obtained from dietary sources [19]: beta-carotene from plants and retinyl esters from animal products such as liver, dairy products, and egg yolk. Retinol is converted in the intestinal epithelium to retinyl esters in chylomicrons, which are then stored or metabolized to retinol and its derivatives in the liver. The retinol-binding protein 4 (RBP4) binds and transports vitamin A to extrahepatic tissues [20].

Kidneys are important organs in maintaining vitamin A homeostasis, as vitamin A bound to serum RBP4 is reabsorbed in 99% in the proximal tubules via endocytic receptor megalin [21,22,23]. An experimental model of megalin deficiency altered vitamin A metabolism by accelerating the mobilization of hepatic vitamin A stores to maintain normal plasma levels [21]. Vitamin A homeostasis is also disturbed in CKD, and patients with impaired kidney function may exhibit elevated circulating retinol, retinoic acid, and RBP4 levels [24]. Frey et al. [25] showed that the occurrence of RBP4 isoforms and their levels are increased in CKD patients, whereas they do not differ between patients with chronic liver disease and healthy controls. This observation may indicate the significance of RBP4 as a marker of kidney function, independent of liver disorders. There are conflicting opinions regarding the relationship between altered vitamin A metabolism and comorbidities such as diabetes and obesity [26]. Abnormal vitamin A and RBP4 levels have been associated with diabetes and insulin resistance, obesity, and dyslipidemia [27]. RBP4 has also been proposed as a novel biomarker for cardiovascular disease due to its association with established cardiovascular risk factors [27,28]. Elevated RBP4 has been linked to major adverse cardiac events in patients with chronic heart failure [29]. The link between vitamin D deficiency and postoperative agitation or other mental status changes after cardiac surgery has been reported [17]. Though retinoid signaling in brain function and mood regulation is important, direct evidence linking perioperative serum retinol levels with postoperative delirium has not yet been demonstrated.

Our findings of a positive correlation between retinol and both preoperative and postoperative creatinine levels are consistent with studies showing higher retinol levels in patients with impaired kidney function. In the CKD subgroup of the National Health and Nutrition Examination Survey (NHANES), serum vitamin A levels were negatively correlated with eGFR (r = −0.56) and positively correlated with creatinine and blood urea nitrogen [30]. Notably, higher serum vitamin A levels were independently associated with all-cause mortality in the CKD population. Increased retinol levels have been suggested to contribute to immune activation [31], potentially explaining both our results and those of other studies.

Several biomarkers have been investigated for early AKI detection and staging scores. Among the proposed molecules, tissue inhibitor of metalloproteinase 2 (TIMP-2), insulin-like growth factor-binding protein 7 (IGFBP7), kidney injury molecule 1 (KIM-1), and neutrophil gelatinase-associated lipocalin (NGAL) have demonstrated high clinical utility [9,32,33]. Urinary RBP showed similar predictive power as more established serum markers [32,34]. NephroCheck, which combines the TIMP2•IGFBP7 panel, demonstrated promising predictive value in cardiac surgery and transcatheter interventions [35,36]; however, the precise thresholds for different intervention models need to be validated. In turn, retinoic acid has protective properties, is involved in the repair of renal tubular injury, and promotes podocyte differentiation [21]. It participates in cellular proliferation, differentiation, and apoptosis [37]. Exogenous pharmacological retinoic acid may enhance the treatment of kidney cancer or diabetic kidney disease [38]. Although experimental studies have focused on retinoic acid, the biologically active metabolite of vitamin A with potential nephroprotective properties, our findings relate specifically to circulating retinol. Retinoic acid acts at the cellular level through nuclear receptors and gene-regulatory effects, and its tissue concentrations are tightly controlled and difficult to measure reliably in routine clinical practice. Though its analysis would be beneficial, we sought to propose markers that would be available in clinical diagnostics.

In particular, identification of markers that are feasible for early recognition of surgery-associated AKI is beneficial for early implementation of therapy [39]. In this context, retinol may possibly represent an additional, readily measurable marker associated with renal vulnerability for AKI occurrence. This hypothesis requires confirmation in larger-scale studies.

Among laboratory data, preoperative renal function parameters, erythrocyte count and hemoglobin differed in patients with postoperative AKI. This likely reflects the influence of baseline renal dysfunction and chronic anemia, as the study group had a high rate of comorbidities, which might have affected the outcome and led to anemia of chronic disease. However, most patients in this study developed only mild postoperative kidney injury, and only two required RRT. The higher proportion of older patients in the AKI subgroup is consistent with previous reports and reflects the age-related decline in renal function [1].

Our findings confirmed previous reports on the existing interaction between retinol and kidney function. The association between higher preoperative retinol concentrations and postoperative AKI may reflect impaired renal handling of retinol-RBP4 complexes rather than a direct causal effect of vitamin A excess. Under physiological conditions, retinol is bound to RBP4 and reabsorbed in the proximal tubules via megalin-mediated endocytosis. Any disturbances in this pathway may lead to altered circulating vitamin A levels. Accordingly, elevated retinol may serve as a marker of early renal vulnerability in patients undergoing cardiac surgery. This interpretation is consistent with previous studies showing altered vitamin A homeostasis in CKD. However, we should also recognize the pro- and anti-inflammatory properties of alterations in vitamin status. The laboratory research by Gans et al. [40] derived proliferative cardiac cells from the human left ventricle, treated them with retinoic acid, and showed increased secretion of inflammatory factors, immune cell activation, and decreased extracellular matrix expression. The authors underlined the surge in cardiac vitamin A metabolism that occurs after heart injury, such as myocardial infarction, resulting from mobilization of vitamin A from peripheral stores and the concomitant upregulation of enzymes involved in retinol metabolism. They stated that in heart injury, retinol may contribute to adverse cardiac remodeling. This study is important in the context of our study setting, as cardiac surgery, as an injury, might also impede mechanisms activating retinol metabolism. We should be aware that increased retinol concentrations in our cohort may identify patients with combined cardiorenal vulnerability. Further experimental studies are necessary to assess this hypothesis.

Our study group represents a real-life, all-comers population of patients undergoing cardiac surgery for CAD and AS. In this setting, our findings may serve as a preliminary report that vitamin status could be a useful parameter in the preoperative risk assessment and prehabilitation. This is particularly relevant in cardiac surgery, CAD, and AS patients, who are burdened with a significant rate of comorbidities, including diabetes, arterial hypertension, COPD, diffuse atherosclerosis, and PAD, all of which may affect kidney function. Moreover, the use of pharmacotherapy for heart failure and coronary artery disease, such as angiotensin-converting enzyme inhibitors and sodium-glucose cotransporter 2 (SGLT2) inhibitors, may influence kidney function in the preoperative and perioperative settings. Currently, there is no targeted therapeutic medication that should be implemented to prevent AKI. Further analyses verifying the medication’s effect on retinol, as well as the uncontrolled use of supplements by patients, are important.

Recent literature has emphasized a patient-centered perspective in cardiac surgery, highlighting both clinical factors, including disease progression and co-morbidities, and psychosocial challenges such as anxiety, depression, and lack of social support as important determinants of postoperative recovery and long-term outcomes [41,42]. In this context, our findings regarding pre-operative kidney function and vitamin status may be interpreted as the need for wide-spectrum prehabilitation, which may improve not only patients’ surgical outcomes and survival, but also quality of life.

Finally, we analyzed vitamin levels in patients with and without diabetes concerning their risk of cardiac surgery-associated AKI. In previous studies, a low 25(OH)3 was associated with a higher risk of AKI and dialysis in the diabetic population [43] or critically ill [44]. The combined effect of CKD and vitamin D deficiency for postsurgical outcomes, including 30-day mortality, AKI, pneumonia, acute myocardial infarction, and atrial arrhythmias, has also been postulated [45]. We did not reveal any significant differences between diabetic and non-diabetic patients in either vitamin D, A, or E. Moreover, we did not observe a significant association between preoperative vitamin D and tocopherol and AKI. Several factors may account for these findings. It should be noted that the majority of our study group had low vitamin D levels, similarly to the general Polish population [46,47]. This issue may affect how we interpret our results and hinder our ability to identify a concentration-related relationship with AKI. Interesting findings come from the meta-analysis by Zhang et al. [48], which suggested that the serum 1,25(OH) vitamin levels, rather than 25(OH) vitamin D, are significantly lower in AKI patients. Thus, more detailed vitamin D subtype analysis [49] may be necessary in these patient groups. Moreover, only mild, mostly transient stages of cardiac surgery-associated AKI predominated in our study, with just two patients requiring temporary renal replacement therapy, which may have reduced the contrast between both groups. Third, our study group was relatively small, so we may have missed weaker associations for vitamins D and E compared with the more robust signal observed for retinol.

While vitamin D levels are more clearly defined in the Polish population and are more commonly examined in daily practice, retinol levels are rarely evaluated. In the study by Godala et al. [50], the mean retinol concentration in the healthy population was 1.8 μmol/L (approx. 0.52 µg/mL) and was even lower in patients with metabolic syndrome (1.37 μmol/L). Similar or only slightly higher values were presented in the analysis of healthy subjects from five European countries [51]. Therefore, our AKI group presents higher values. In our cohort, the median retinol concentration was 0.64 mcg/mL, and the mean retinol concentration in patients with AKI was 0.7 mcg/mL, which is the upper reference limit for high retinol levels. In non-AKI patients, the median was 0.56 mcg/mL, and the mean was 0.59 mcg/mL. Therefore, retinol concentrations in the AKI group were at the upper end of, or above, the reference range.

Consistent with previous surgical series [52,53,54], our cohort showed a male predominance among patients undergoing coronary artery and valvular surgical procedures. This observation likely reflects the earlier onset of coronary artery disease and degenerative aortic stenosis in men of operative age, and delays in operative interventions in females due to the fact that women often present later and are referred for surgery at an older age with a higher risk profile [55]. These factors are also related to the prevalence of AKI [56]. A meta-analysis [57] of 64 studies showed that females are more prone to develop AKI than males. However, further analysis indicated a strong dependence on the higher age and co-morbidities of females at the time of surgery for AKI occurrence. According to Demirjian et al. [58], the definition of pre-operative kidney function and misclassification due to AKI, as defined by absolute change in creatinine, may lead to misleading outcomes evaluation.

Moreover, in sex-stratified analyses, we observed a statistically significant difference in preoperative 25(OH)D3 concentrations, with women having lower vitamin D than men, and sex explained only 7% of the variance in 25(OH)D3. In contrast, sex was not significantly associated with serum retinol concentrations and showed only a small, non-significant trend towards lower tocopherol levels in women, with sex accounting for 4% of the variance in vitamin E. Overall, these findings suggest that, in our study group, sex had a trivial to modest influence on vitamin status, and other factors are probably more likely to determine their concentrations.

### 4.1. Future Perspective

In the context of the emerging “twin transformation” in cardiothoracic surgery, which combines digital innovation with sustainability-oriented care models [59], improved risk stratification for cardiac surgery-associated AKI using clinical variables and biomarkers may contribute to more precise and clinically efficient perioperative pathways. A recent study by Pylarinou et al. [60] introduced a clinical decision support system specifically designed for post-surgical cardiovascular remote monitoring, integrating patient history, intraoperative data, monitoring streams, and surgical outcomes into an artificial intelligence-based architecture that may guide clinical recommendations. The implementation of artificial intelligence-assisted decision-making tools, the earlier identification of patients at higher risk of post-operative complications, including even a mild degree of AKI, and targeted modifications to improve outcomes by reducing the complication rate, the need for RRT, and prolonged intensive care stay.

### 4.2. Limitations

This study has several limitations. Firstly, in our study, serum or urine RBP4 was not measured, which limited the assessment of vitamin A transport and metabolism. Secondly, the study population was burdened by several comorbidities, which may limit generalizability. Finally, given that the patient group was relatively small, particularly with respect to severe AKI and RRT use, and the borderline statistical significance for retinol (*p* = 0.046), and the exploratory nature of the study, the present finding should be treated as hypothesis-generating.

## 5. Conclusions

Retinol may be associated with increased vulnerability to cardiac surgery-associated AKI. However, its clinical utility as a biomarker requires validation in larger cohorts.

## Figures and Tables

**Table 1:** Baseline demographic and clinical characteristics.

	All Patients (*n* = 87)	Patients with AKI (*n* = 36)	Patients Without AKI (*n* = 51)	*p*
Age	66 (61.5–71)	68.5 (64–73)	66 (60–70)	0.025
Male sex (n, %)	72 (82.8)	29 (80.6)	43 (84.3)	0.78
BMI (kg/m^2^, median, Q1–Q3)	28.4 (25.6–31.4)	28.6 (26.2–31.1)	28.3 (24.9–32.2)	0.73
HA (n, %)	73 (83.9)	31 (86.1)	42 (82.4)	0.77
DM/IGT (n, %)	32 (36.8)	16 (44.4)	16 (31.4)	0.21
Only DM (n, %)	28 (32.2)	15 (41.7)	13 (25.5)	0.11
AF (n, %)	10 (11.5)	4 (11.1)	6 (11.8)	1.00
COPD/asthma (n, %)	7 (8.1)	2 (5.6)	5 (9.8)	0.70
History of MI (n, %)	18 (20.9)	6 (16.7)	12 (24)	0.59
History of PCI (n, %)	12 (14.0)	5 (13.9)	7 (14)	1.00
Previous stroke or TIA (n, %)	36 (6.0)	2 (5.6)	4 (8.0)	1.00
PAD (n, %)	14 (16.3)	8 (22.2)	6 (12.0)	0.24
CPB (n, %)	25 (28.7)	8 (22.2)	17 (33.3)	0.34
Preoperative creatinine (umol/L, median, Q1–Q3)	0.90 (0.76–1.02)	0.95 (0.82–1.15)	0.87 (0.73–0.98)	0.01
Preoperative eGFR (mL/min, median, Q1–Q3)	85.2 (68.1–99.1)	68.5 (59.3–89.4)	87.8 (74.4–103.2)	0.002
Preoperative eGFR < 60 mL/min (n, %)	14 (16.1)	11 (30.6)	3 (5.9)	0.002
Preoperative leucocyte count (×10^9^/L, median, Q1–Q3)	7.4 (6–8.6)	7.5 (6.3–8.3)	7.1 (5.8–8.8)	0.97
Preoperative haemoglobin (mmol/L, median, Q1–Q3)	8.6 (8.2–9.3)	8.4 (8.1–9.1)	8.8 (8.4–9.5)	0.035
Preoperative erythrocyte count (×10^12^/L, median, Q1–Q3)	4.7 (4.4–5.0)	4.6 (4.3–4.8)	4.8 (4.5–5.1)	0.04
Preoperative platelet count (×10^9^/L, median, Q1–Q3)	230 (193–269)	228 (188–253)	230 (197–292)	0.26
Preoperative NTproBNP (pg/mL, median, Q1–Q3)	427.7 (184.1–570.1)	462.6 (435.5–605.9)	186.9 (106.9–432.3)	0.19
ALT (U/L, median, Q1–Q3)	33.0 (21.0–44.3)	33.5 (23.3–41.3)	32.0 (21.0–47.3)	0.84
AST (U/L, median, Q1–Q3)	27.0 (22.8–37.0)	28.0 (24.3–40.50	27 (22.0–34.8)	0.41
Postoperative 0 day Creatinine (umol/L, median, Q1–Q3)	1.04 (0.92–1.22)	1.23 (1.03–1.42)	0.93 (0.84–1.07)	<0.001
Postoperative 1 day Creatinine (umol/L, median, Q1–Q3)	1.12 (0.88–1.39)	1.43 (1.22–1.75)	0.93 (0.80–1.11)	<0.001
Postoperative 2 day Creatinine (umol/L, median, Q1–Q3)	0.98 (0.78–1.27)	1.26 (1.09–1.59)	0.84 (0.68–1.00)	<0.001
Retinol (mcg/mL, median, Q1–Q3)	0.59 (0.48–0.75)	0.64 (0.56–0.83)	0.56 (0.45–0.72)	0.046
25(OH)D3 (ng/mL, median, Q1–Q3)	14.4 (6.1–27.2)	15.0 (7.6–28.2)	10.2 (5.7–25.2)	0.37
Tocopherol (mcg/mL, median, Q1–Q3)	12.7 (9.7–16.5)	13.4 (9.6–16.9)	12.5 (9.9–15.7)	0.33

Abbreviations: AKI—acute kidney injury; AF—atrial fibrillation; ALT—alanine aminotransferase; AST—aspartate aminotransferase; BMI—body mass index; COPD—chronic obstructive pulmonary disease; CPB—cardiopulmonary bypass; DM—diabetes mellitus; eGFR—estimated glomerular filtration rate; IGT—impaired glucose tolerance; HA—arterial hypertension; MI—myocardial infarction; NTproBNP—N-terminal pro-B-type natriuretic peptide; PAD—peripheral arterial disease; PCI—percutaneous coronary intervention; TIA—transient ischemic attack.

**Table 2:** Vitamin levels in patients with and without DM, presenting with or without AKI.

	DM(+)AKI(+)	DM(+)AKI(−)	DM(−)AKI(+)	DM(−)AKI(−)	*p*
Retinol (mcg/mL, median, Q1–Q3)	0.69 (0.57–0.87)	0.56 (0.46–0.76)	0.63 (0.54–0.73)	0.56 (0.45–0.71)	0.20
25(OH)D3 (ng/mL, median, Q1–Q3)	14.7 (5.6–24.0)	9.9 (6.2–18.1)	17.0 (9.0–31.1)	11.5 (5.6–32.3)	0.41
Tocopherol (mcg/mL, median, Q1–Q3)	12.8 (9.4–16.8)	13.9 (13.0–17.8)	13.9 (11.1–16.9)	11.3 (9.6–15.0)	0.23

Abbreviations: AKI—acute kidney injury; DM—diabetes mellitus. (+) presence; (−) absence.

## Data Availability

The original contributions presented in this study are included in the article/Supplementary Material. Further inquiries can be directed to the corresponding author.

## Supplementary Materials

The following supporting information can be downloaded at https://www.mdpi.com/article/10.3390/nu18121921/s1.

## Abbreviations

The following abbreviations are used in this manuscript:

AKI	acute kidney injury
AF	atrial fibrillation
ALAT	alanine aminotransferase
AS	aortic stenosis
AspAT	aspartate aminotransferase
AVR	aortic valve replacement
BMI	body mass index
CABG	coronary artery bypass grafting
CAD	coronary artery disease
CKD	chronic kidney disease
COPD	chronic obstructive pulmonary disease
CPB	cardiopulmonary bypass
DM	diabetes mellitus
eGFR	estimated glomerular filtration rate
IGT	impaired glucose tolerance
HA	arterial hypertension
HF	heart failure
MI	myocardial infarction
MDRD	Modification of Diet in Renal Disease
NTproBNP	N-terminal pro-B-type natriuretic peptide
PAD	peripheral arterial disease
PCI	percutaneous coronary intervention
RRT	renal replacement therapy
TIA	transient ischemic attack
