# Supplementary material for: Increased Retinol Levels in Patients with Cardiac Surgery-Associated Acute Kidney Injury—A Prospective Single-Center Exploratory Study
Source: Nutrients. 2026 Jun 13;18(12):1921. doi: 10.3390/nu18121921 (PMC13304596; doi:10.3390/nu18121921)
Supplement: Supplementary file 1 [file nutrients-18-01921-s001.zip › nutrients-4362613-supplementary.pdf]

Category	Major risk factors	Minor / additional risk factors
Patient-related	Advanced age; pre-existing chronic kidney disease; diabetes mellitus; heart failure; low preoperative eGFR or elevated creatinine; high surgical risk scores (e.g. EuroSCORE II)	Female sex; peripheral arterial disease; chronic obstructive pulmonary disease; anemia; malnutrition (low prognostic nutritional index)
Procedural (intraoperative)	Use and duration of cardiopulmonary bypass; prolonged aortic cross-clamp time; complex or combined procedures (e.g. CABG + valve surgery); intraoperative hypotension and low cardiac output	Hemodilution; large volume transfusion; use of nephrotoxic drugs; robotic/long operative time
Postoperative	Persistent low cardiac output; vasopressor requirement; sepsis or severe infection; hemodynamic instability; need for mechanical circulatory support	Fluid overload; exposure to additional nephrotoxic agents; prolonged mechanical ventilation
